# Multiplexed video hyperspectral microendoscopy reveals tumor–immune dynamics in vivo

**DOI:** 10.21203/rs.3.rs-9856874/v1

**Published:** 2026-06-22

**Authors:** Bryan Spring, Mohammad Saad, Rebecca Harman, Eric Kercher, Ryan Lang, Kai Zhang, Julia Tatz, Qianqian Fang, Jason Sutin, Zhiming Mai, Akilan Palanisami, Tayyaba Hasan

**Affiliations:** Northeastern University; Massachusetts General Hospital; Northeastern Univerisity; University of Massachusetts; Northeastern Univerisity; Northeastern Univerisity; Boston Children's Hospital; Northeastern University; Boston Children's Hospital; Massachusetts General Hospital; Massachusetts General Hospital; MGH, Harvard Medical School and HST

## Abstract

Tumor–immune interactions evolve dynamically within heterogeneous tumor microenvironments, yet methods for repeatedly measuring multiple cellular phenotypes *in vivo* remain limited. We developed multiplexed video hyperspectral microendoscopy to image fluorescently labeled tumor and immune cell biomarkers in living tissue at cellular resolution. The platform combines photon-efficient spectral detection with rapid spectral unmixing to resolve cocktails of antibody and ligand probes and quantify multiple tumor and immune biomarkers simultaneously *in vivo*. In mouse tumor models, longitudinal imaging revealed spatially organized tumor and immune cell phenotypes and quantified treatment-associated changes within individual animals. Wide-field mosaics extended the microendoscopic field of view to map biomarker heterogeneity across tumors. These results establish video hyperspectral microendoscopy as a platform for longitudinal measurement of spatially structured tumor–immune dynamics *in vivo*.

Spatial and phenotypic heterogeneity within malignant lesions drives therapeutic resistance and variability in patient outcomes (1). Stem-like states, immune–tumor crosstalk, proliferative gradients, and metabolic diversity contribute to treatment failure (1, 2) and are often spatially organized within the tumor microenvironment. Advanced *ex vivo* approaches—including multiplexed immunofluorescence (3, 4), imaging mass cytometry (5, 6), and lineage barcoding (7)—have substantially improved our understanding of microscale tumor architecture and its relationship to clinical outcomes. However, identifying rare microenvironmental niches defined by specific biomarker combinations that promote treatment escape remains challenging (8). Moreover, because biopsy-based sampling provides only static snapshots of limited tissue regions, the spatiotemporal evolution of tumor–immune phenotypes during therapy—including the emergence of resistant cell states and targetable biomarkers that could inform personalized treatment—remains difficult to monitor *in vivo*.

Intravital optical microscopy enables cellular-resolution imaging in living tissues, but multiplexed molecular phenotyping at video rate remains challenging. Video-rate acquisition is essential for minimizing motion artifacts arising from respiration, cardiac pulsation, and mechanical instability of handheld or fiber-based probes. However, prior multispectral video intravital microscopy and fiber microendoscopy systems have typically resolved no more than two molecular markers simultaneously (9–12), limiting the ability to interrogate complex tumor–immune interactions. Extending multiplexing to video-rate acquisition introduces a fundamental physical constraint—at cellular resolution and physiological excitation levels, fluorescence emission is frequently shot-noise limited. Under these photon-limited conditions, short integration times impair spectral estimation accuracy, complicating reliable separation of overlapping fluorophores without quantitative calibration.

Here we introduce Spectral Multiplexed *In vivo* Real-time Imaging with Cellular resolution (SMIRC), a multiplexed spectral fiber microendoscopy platform for quantitative, video-rate phenotypic mapping of the tumor microenvironment. SMIRC integrates 15-channel contiguous spectral detection with experimentally calibrated variance–mean photon analysis and high-speed spectral unmixing, enabling simultaneous imaging of five tumor and immune biomarkers using multiplexed fluorophore-conjugated probes at 17 spectral image cubes per second. We apply this approach to xenograft models of peritoneal metastases and to an immunocompetent syngeneic mouse model of pancreatic adenocarcinoma to demonstrate multiplexed visualization of tumor and tumor-infiltrating immune cell phenotypes and longitudinal assessment of treatment-associated changes. Because conventional *ex vivo* biopsy samples only small and potentially unrepresentative tissue regions, *in vivo* microendoscopy enables scanning across larger tumor areas to reduce sampling bias. Wide-field microimage mosaics were therefore generated to expand the effective field of view and enable quantitative mapping of biomarker spatial heterogeneity across tumor regions.

Compared with *ex vivo* vibratome-based tissue slicing (13) and mass spectrometry imaging (14), which capture single time points, fiber-based microendoscopy enables repeated interrogation of regions within the same lesion over time. Intravital multiphoton microscopy provides high spatial resolution but typically requires surgical window implantation and acquisition times on the order of seconds per frame (15, 16), limiting its suitability for video-rate multiplexing. In contrast, SMIRC acquires spectral image cubes in ~ 60 ms (17 Hz), enabling video-rate sampling of multiplexed tumor and immune phenotypes within anatomical sites accessible to fiber probes. By combining quantitative spectral separation with longitudinal *in vivo* access, multiplexed spectral microendoscopy provides a framework for resolving spatially organized tumor–immune states and their evolution during therapy in preclinical cancer models.

## Photon-calibrated multiplexed spectral imaging at video rate

**Figure 1:**
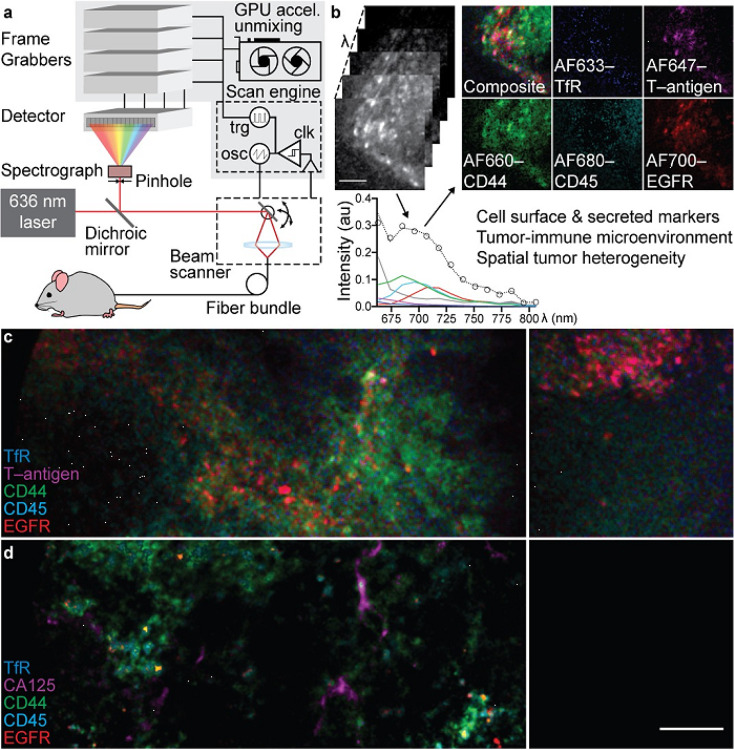
SMIRC instrumentation and in vivo multiplexed imaging. (**A**) Schematic of the SMIRC platform generating spectral image cubes spanning emission wavelengths at 16.7 Hz (~60 ms per frame). (**B**) Spectral image stack decomposition by linear unmixing to recover abundance maps of fluorescent targeting conjugates. Example data from a live orthotopic pancreatic tumor. (**C**) Five-biomarker multiplexed SMIRC imaging of an orthotopic pancreatic tumor (left) and a nearby peritoneal metastasis (right). (**D**) Five-biomarker multiplexed SMIRC imaging of peritoneal carcinomatosis (left) and a no-tumor control mouse (right). Scale bars, 100 μm. AF, Alexa Fluor; TfR, transferrin receptor.

SMIRC spectral detection was designed using compressive sampling principles (17) to enable efficient estimation of pixel spectra from low-abundance fluorescence signals (Fig. 1A and B; figs. S1–S3). A GPU-accelerated fast non-negative least squares (GPU-FNNLS) algorithm enabled high-speed spectral unmixing at rates exceeding acquisition (> 17 Hz; processed offline; figs. S4–S7). *In vitro* imaging of fixed cancer cells demonstrated cellular-resolution detection of five to six molecular markers simultaneously (figs. S8 and S9). Goodness-of-fit analysis using defined fluorophore mixtures (0.1–10 μM) confirmed accurate identification of up to three biomarkers per cell type using a five-fluorophore basis library, and demonstrated the benefit of staggered labeling across cell populations to minimize spectral overlap (figs. S10–S13).

Representative *in vivo* multiplexed imaging of pancreatic tumors and peritoneal metastases are shown in Fig. 1 (C and D), demonstrating simultaneous five-biomarker spectral decomposition at video rate in orthotopic mouse models.

**Figure 2:**
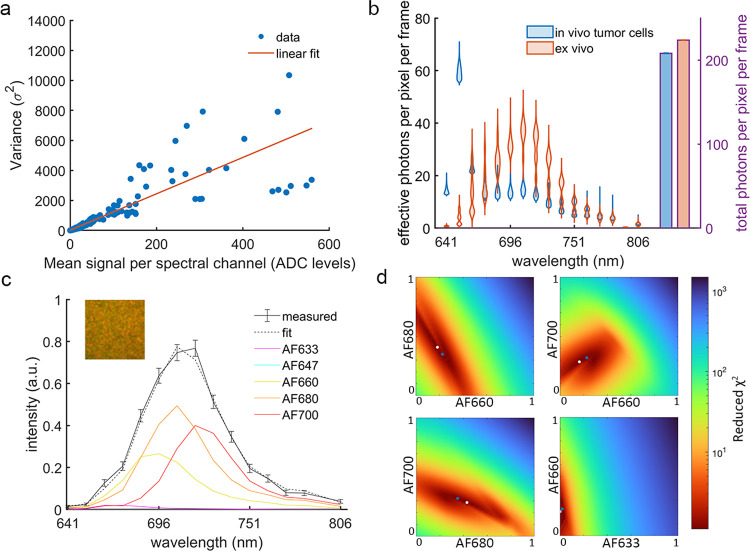
Photon-limited calibration and statistical validation of multiplexed spectral unmixing. (**A**) Variance–mean analysis demonstrating photon-limited detection. SMIRC images of Alexa Fluor 660 (10 μM) acquired across excitation powers were analyzed to compute pixel-wise variance as a function of mean fluorescence intensity. Variance scaled linearly with mean signal as σ^2^ = (10.0 ± 0.3) μ + (2.9 ± 2.7), R^2^ = 0.986 within the low-signal regime relevant to *in vivo* imaging, consistent with shot-noise–limited detection. Signal values are reported in ADC units (10-bit range). Spectral channels were analyzed independently without unmixing. The calibrated variance–mean relationship was then used to estimate effective photon counts per pixel *in vitro* and *in vivo*. (**B**) Comparison of effective photon counts *in vitro* and *in vivo*. Effective photons per processed pixel per frame were estimated using the calibrated variance–mean relationship. Left, per-channel photon distributions shown as violin plots. Right, total effective photon counts for fluorophore mixtures in solution and representative in vivo tumor cells (mean ± s.e.m.; n = 10 images per condition). Overall photon budgets were similar between *in vitro* and *in vivo* conditions, indicating that fluorophore-in-solution experiments approximate in vivo signal levels under video-rate acquisition. (**C**) Spectral unmixing of a three-dye mixture using a five-dye basis library. Fluorophores AF660, AF680, and AF700 were mixed in solution and imaged with SMIRC. Spectral decomposition was performed using a five-fluorophore basis set (AF633, AF647, AF660, AF680, AF700) plus PBS background. Representative unmixed image (110 μm × 110 μm) and center-pixel spectral fit. Measured emission (solid black) and GPU-accelerated non-negative least-squares fit (dashed black) demonstrate accurate reconstruction. Basis contributions are shown for each fluorophore. The reduced chi-square value was = 1.14 (9 degrees of freedom; 15 spectral channels, 6 basis spectra), indicating that residuals were consistent with expected photon (shot) noise and adequate spectral model fit. (**D**) Reduced landscapes for spectrally overlapping fluorophores. Pairwise exemplary heatmaps for AF633, 660, 680 and 700 contributions corresponding to (C). Basis coefficients for the dyes were systematically varied and recalculated. The FNNLS best-fit solution is indicated (blue dot), and the global minimum is marked (white dot), demonstrating well-defined parameter convergence despite spectral overlap.

To define the photon-limited operating regime, variance–mean analysis was performed on uniform fluorophore images acquired across excitation powers. Variance scaled linearly with mean intensity, consistent with shot-noise–limited detection (Fig. 2A). Calibration yielded ~ 200 total photons per pixel per frame (~ 10 photons per spectral channel; Fig. 2B). Mean signal levels per spectral channel were comparable between uniform dye solutions and representative *in vivo* tumor regions acquired at video rate (Fig. 2B), indicating that dye measurements approximate *in vivo* photon budgets while avoiding spatial heterogeneity. Applying the same calibration to *in vivo* data confirmed photon-limited operation (Fig. 2B). Reliable molecular identification was achieved by integrating spatially and temporally oversampled measurements and pooling information across spectrally distinct channels during unmixing. Representative spectral unmixing of a three-dye mixture using a five-dye basis set demonstrated accurate reconstruction with reduced *χ*^2^ values near unity under photon-limited conditions (Fig. 2C and D).

### In vivo multiplexed phenotyping of tumor and immune markers

To evaluate *in vivo* performance, orthotopic xenograft models of pancreatic cancer (AsPC1 cell line) (18) and peritoneal metastases (19) (OVCAR5 cell line) were imaged using a handheld fiber microendoscope (Fig. 1C and D). Fluorophore-conjugated antibodies and ligands targeting cell-surface receptors or extracellular matrix–associated proteins were administered intraperitoneally 1 hour prior to imaging, and minimally invasive imaging was performed via catheter access.

Multiplexed imaging resolved biomarkers associated with cancer cell proliferation and survival (EGFR) (19–21), stem-like or mesenchymal phenotypes (CD44) (22–24), tumor-associated glycoproteins (CA125/MUC16) (25–28), transferrin receptor activity (29), pan-carcinoma glycan expression (T antigen) (30), and immune infiltration (CD45) (31). In typical imaging configurations, combinations of five biomarkers were resolved simultaneously, enabling identification of distinct tumor cell phenotypes and immune populations at single-cell resolution. Targeted conjugates produced 5–15× higher signal than non-specific isotype controls in two mouse models (fig. S14). *Ex vivo* histology and immunofluorescence of the OVCAR5 model of peritoneal metastases confirmed heterogeneous tumor expression and negligible signal in no-tumor controls (figs. S15 and S16). Single-cell–resolved phenotypes were routinely observed *in vivo*.

## Longitudinal quantification of tumor-infiltrating T cells

**Figure 3:**
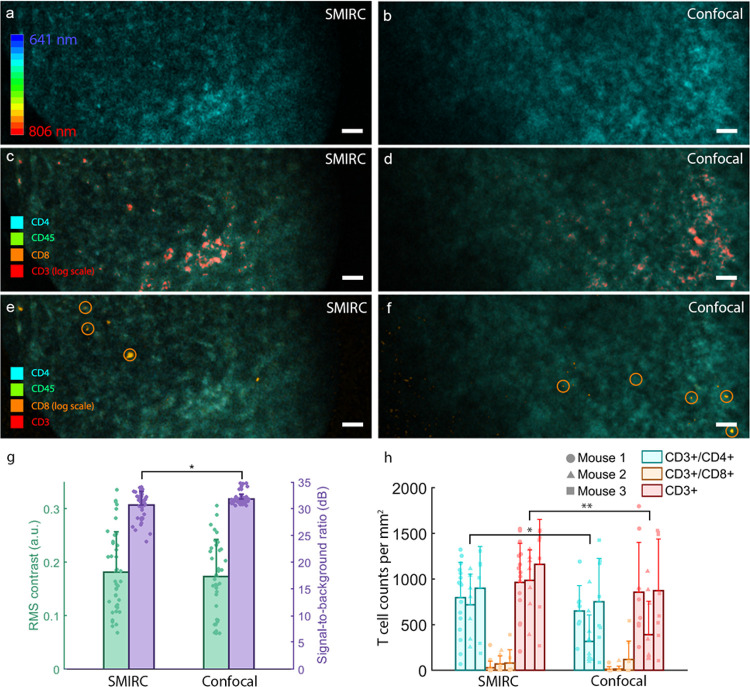
Quantitative benchmarking of SMIRC against direct confocal imaging. Freshly excised pancreatic tumors from immunocompetent KPC mice were imaged following intraperitoneal administration of antibody–fluorophore conjugates. (**A** and**B**) Representative raw spectral images pseudocolored by detection channel for SMIRC imaging through the coherent fiber bundle at 17 Hz (A) and for direct confocal imaging using the identical optical system without the fiber bundle (B). Full fields of view are shown. In SMIRC images, peripheral dark regions correspond to the physical boundary of the fiber bundle. In direct confocal images, peripheral dark regions arise from local variations in tissue surface height relative to the confocal focal plane. Scale bars, 50 μm. (**C** and **D**) Corresponding spectrally unmixed composite images with the CD3+ channel displayed on a logarithmic scale to enhance visualization of sparse T cells. Spectral unmixing was performed using identical reference spectra and processing pipelines for both modalities. Scale bars, 50 μm. (**E** and**F**) Spectrally unmixed CD8 channel images (log scale). Circles denote representative CD3+/CD8+ cells. Scale bars, 50 μm. (**G** and **H**) Quantitative comparison of SMIRC and confocal imaging. (G) Image quality metrics for the unmixed CD4 channel following background segmentation, including root-mean-square contrast and maximum signal-to-background ratio. (H) Immune cell densities (cells per mm^2^) for total CD3+, CD3+/CD4+, and CD3+/CD8+ T cells. Data are mean ± s.d.; points represent individual fields of view (n = 3 mice; 7–12 images per mouse per modality). Statistical comparisons were performed using unpaired two-tailed Student’s *t*-tests (*P£0.05, **P£0.01). Cell counts were obtained using a custom CellProfiler pipeline and normalized by effective imaging area for fiber-based measurements.

To assess immune profiling capability, fluorophore-conjugated antibodies targeting CD3, CD4, CD8, and CD45 were administered in the KPC (*KrasLSL.G12D*/+; *p53R172H*/+; *PdxCretg*/+) syngeneic immunocompetent model of pancreatic ductal adenocarcinoma (32). Quantitative benchmarking of SMIRC against direct confocal imaging without the fiber bundle demonstrated preservation of image quality metrics and immune cell densities across modalities (Fig. 3A–H). Freshly excised tumors were imaged *ex vivo* using both fiber-based SMIRC and direct confocal imaging without the fiber bundle under identical optical conditions. Although fiber imaging modestly reduced contrast and signal-to-background ratio, quantitative T cell densities (Fig. 3H) and processed single-pixel spectra were comparable across modalities (fig. S17), indicating preservation of quantitative immune measurements.

**Figure 4:**
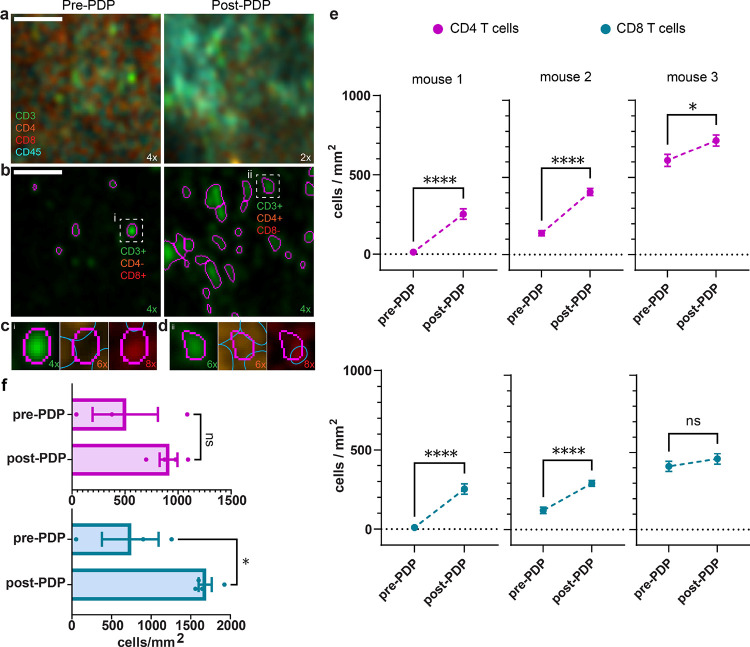
Longitudinal in vivo quantification of tumor-infiltrating T cells with ex vivo validation following photodynamic priming. (**A**) Representative spectrally unmixed composite SMIRC images of an immunocompetent KPC pancreatic ductal adenocarcinoma tumor imaged *in vivo* before (pre-PDP) and 4 days after verteporfin photodynamic priming (post-PDP). Increased CD3+ signal is observed post-treatment. Display brightness was uniformly scaled (2–4×) for visualization. Scale bar, 50 μm. (**B**) CD3 channel with identified CD3+ cells (magenta outlines) for the images in (A), demonstrating increased tumor-infiltrating T cells post-PDP. Cells were identified using a custom CellProfiler pipeline (*Supplementary Methods*). Scale bar, 50 μm. (**C** and **D**) Representative zoomed views of identified CD8+ (c; CD3+/CD4-/CD8+; image dimension, 18.7 μm) and CD4+ (d; CD3+/CD4+/CD8-; image dimension, 26.4 μm) T cells. Magenta outlines indicate classified cells; blue outlines denote CD3-cells positive for CD4 or CD8. (**E**) In vivo SMIRC quantification of CD3+/CD4+(top) and CD3+/CD8+ (bottom) T cells (cells per mm^2^) in individual mice pre- and post-PDP (n = 3 mice; 10–22 non-overlapping fields per mouse per time point). CD4+ T cells increased in all mice (P < 0.0001 for mice 1–2; P < 0.05 for mouse 3, Welch’s *t*-test). CD8+ T cells increased significantly in two mice (P < 0.0001, Welch’s *t*-test). Data are mean ± s.e.m. (**F**) *Ex vivo* immunofluorescence quantification of CD3+/CD4+ and CD3+/CD8+ T cells at the tumor edge from whole-tumor cross-sections (pre-PDP: *n* = 3 mice; post-PDP: *n* = 4 mice). CD8+ T cells increased significantly post-PDP (P < 0.05, unpaired Student’s *t*-test); CD4+ T cells showed an increasing trend. Data are mean ± s.e.m.

Photodynamic priming (PDP), a non-ablative effect of photodynamic therapy that extends beyond the directly cytotoxic zone, was applied using verteporfin and 690 nm light activation to modulate the tumor microenvironment and promote immune cell trafficking (33, 34). The tumor periphery was sampled *in vivo* before and 96 h after PDP. CD3+ lymphocytes were identified by colocalization analysis using CD3/CD4 and CD3/CD8 marker combinations, and quantified using a custom CellProfiler pipeline (Supplementary Methods). Baseline CD3+/CD4+ and CD3+/CD8+ counts were sparse (Figs. 3H and 4A–F), consistent with prior characterization of the KPC model (35). Following PDP, repeated measurements in individual mice revealed increased tumor-infiltrating CD4+ and CD8+ T cells, with inter-animal variability (Figs. 4A–E). *Ex vivo* whole-tumor section immunofluorescence confirmed similar trends (Fig. 4F; figs. S18–S24). *In vivo* and *ex vivo* fold changes in T cell numbers were comparable (CD4+: ~1.8×; CD8+: ~2×), demonstrating longitudinal quantification of immune cell subtype dynamics in individual subjects.

## Wide-field mosaics reveal multiscale spatial heterogeneity

**Figure 5:**
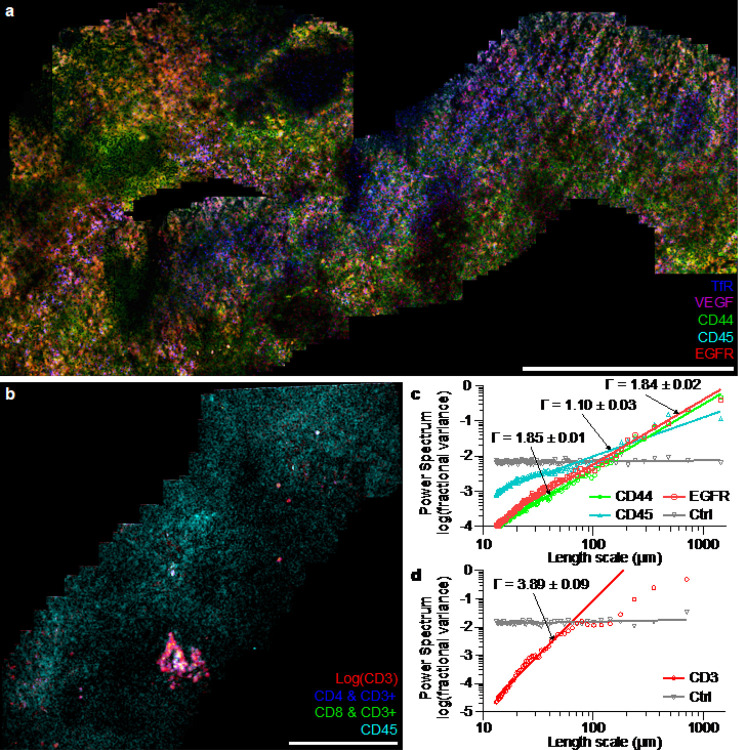
Wide-field SMIRC mosaics and power spectral analysis of spatial heterogeneity. (**A**) Five-biomarker SMIRC microimage mosaic of xenograft peritoneal metastasis. Scale bar, 1 mm. Individual spectrally unmixed channels corresponding to this mosaic are shown in fig. S30. (**B**) SMIRC microimage mosaic of two T cell phenotypes in the syngeneic KPC pancreatic ductal adenocarcinoma model. CD3 signal is displayed on a logarithmic scale to enhance visualization of sparse T cells. Scale bar, 0.5 mm. (**C**) Fourier power spectral analysis of EGFR, CD44, and CD45 intensity distributions in (A). Spectral slopes (G) quantify spatial heterogeneity; solid lines denote power-law fits. The slope for EGFR differs significantly from CD45 (*P*<0.0001, *F*-test of G) but not from CD44 (*P*=0.5938). (**D**) Power spectral analysis of CD3+ T cell clustering in (B), demonstrating strong spatial correlation over intermediate length scales.

To extend microscale imaging across larger areas, computational micro-spectroscopic mosaicking (36) was used to assemble wide-field maps in xenograft peritoneal metastases (Fig. 5A; figs. S25–S29; video S1) and the KPC model (Fig. 5B). Video mosaicking extended the effective field of view from ~ 0.3 mm^2^ per frame to several mm^2^ while preserving cellular resolution, enabling quantitative analysis of tumor spatial heterogeneity.

Fourier power spectrum analysis (37) was applied to characterize spatial organization. The spectral slope (Γ) quantifies how intensity fluctuations scale with spatial frequency, with higher values indicating greater long-range spatial correlation. In xenograft metastases, Γ = 1.84 ± 0.02 for EGFR and Γ = 1.85 ± 0.01 for CD44, indicating pronounced spatial organization (Fig. 5C). In contrast, CD45 exhibited a reduced slope (Γ = 1.10 ± 0.03), consistent with comparatively weaker long-range heterogeneity. In the KPC model, CD3+ T cells demonstrated strong clustering (Γ = 3.89 ± 0.09) over length scales up to 78 μm (Fig. 5D), beyond which the spectrum flattened due to localized T cell aggregates.

## Automated phenotypic mapping and spatial relationship analysis

An automated phenotype-mapping algorithm was developed for SMIRC datasets (fig. S31), generating spatial maps of cancer cell subsets and immune populations and enabling extraction of population frequencies and pairwise distance metrics.

In the OVCAR5 xenograft model of peritoneal metastases, distinct cancer cell subpopulations defined by combinatorial biomarker expression were identified (fig. S31A and B). Following verteporfin PDP, overall cancer cell burden decreased without preferential depletion of a specific phenotypic subset (fig. S31B). Spatial proximity analysis revealed preferential localization of CD44-high cancer cells near CD45+ immune cells relative to CD44-low cells (fig. S31C). For the syngeneic KPC model, exemplary bright, intensity thresholded CD3+/CD4 + and CD3+/CD8 + T cells are shown pre- and post-PDP (fig. S31D). These analyses demonstrate the ability of multiplexed spectral microendoscopy to quantify dynamic cancer–immune spatial relationships *in vivo*.

## Discussion

SMIRC establishes a quantitative framework for real-time, cellular-resolution phenotyping of tumor and immune biomarkers *in vivo*. By integrating photon-calibrated spectral detection, GPU-accelerated unmixing, and fiber-based microendoscopy, the platform operates within a shot-noise–limited regime while preserving reduced *χ*^2^ values near unity during video-rate spectral decomposition. This engineering advance enables multiplexed molecular imaging under physiologic photon budgets, supporting simultaneous detection of five biomarkers and identification of multiple tumor and immune phenotypes at cellular resolution, addressing a central challenge in extending spectral imaging to handheld and deep-tissue configurations.

The ability to resolve up to three fluorophores per pixel using a five-dye basis library reflects the statistical constraints imposed by photon-limited detection and overlapping emission spectra. Rather than relying on high photon flux, SMIRC leverages spatial and temporal oversampling together with constrained spectral fitting to achieve robust molecular separation. In practice, this enables reliable identification of up to three biomarkers per individual cell while resolving five molecular markers within the imaging field. This quantitative validation distinguishes the present approach from prior video-rate systems limited to two markers and provides a principled basis for multiplexed imaging *in vivo*.

Fiber-based imaging inherently introduces trade-offs in contrast and axial resolution relative to conventional confocal or multiphoton microscopy. However, benchmarking against direct confocal imaging demonstrated preservation of quantitative immune cell densities and spectral profiles, indicating that coherent fiber bundle transmission does not compromise cellular-level phenotyping. The reduced axial resolution of the current semi-confocal configuration may limit accurate segmentation in densely packed regions. Future implementations incorporating microlens objectives, improved optical sectioning, or multiphoton excitation architectures may further enhance axial confinement and resolution (11, 38).

Longitudinal monitoring of CD4 + and CD8 + T cell populations following PDP illustrates the capacity of SMIRC to quantify immune dynamics within individual subjects. The observed increases in tumor-infiltrating lymphocytes are consistent with PDP-mediated modulation of the tumor microenvironment (33, 39) and demonstrate that multiplexed spectral microendoscopy can extract immunoscore-like metrics (40) *in vivo*. Because imaging spans larger tissue regions than conventional biopsy-based histopathology, repeated interrogation of the same general lesion areas can be performed without strict spatial re-registration to an identical microscopic field, enabling more comprehensive longitudinal assessment of treatment-induced remodeling.

Wide-field microimage mosaicking combined with Fourier power spectral analysis further revealed multiscale spatial organization of cancer-associated biomarkers and immune cell distributions. The spectral slope (Γ) provided a quantitative measure of long-range heterogeneity, distinguishing clustered tumor markers from more uniformly distributed immune populations. Such spatial statistics offer a complementary dimension to cell density measurements, enabling assessment of spatially structured tumor–immune states that may influence therapeutic response.

Several limitations merit consideration. The current implementation relies on exogenous fluorescent conjugates and is restricted to surface-accessible or probe-accessible anatomical sites. Antibody penetration and optical scattering further constrain imaging depth, limiting measurements primarily to tumor periphery regions accessible to fiber-based microendoscopy. While this restricts sampling of the tumor core, the tumor margin is increasingly recognized as a biologically active interface where immune infiltration, stromal remodeling, and treatment-induced changes in the tumor microenvironment are concentrated. Consistent with this, longitudinal multiplexed imaging from the TONIC trial—derived from serial *ex vivo* biopsy imaging of metastatic lesions—identified spatial features at tumor borders, particularly immune diversity and T cell infiltration, as among the strongest predictors of response to immune checkpoint inhibition (41). These findings suggest that periphery-focused measurements can capture clinically relevant tumor–immune dynamics, while highlighting the opportunity for approaches such as SMIRC to extend such analyses to real-time, *in vivo* longitudinal imaging without repeated tissue sampling.

In addition, while antibody–fluorophore doses were substantially below therapeutic levels (20), formal safety and pharmacokinetic studies will be required for clinical translation, analogous to phase I/II evaluations performed for fluorescence-guided surgery agents (20, 42–46). Multiplexing capacity also remains constrained by the available photon budget and spectral overlap under *in vivo* conditions, motivating continued development of brighter probes, optimized spectral spacing, and improved detection efficiency. The present implementation represents only one realization of a broader hyperspectral microendoscopy framework. Although the current experiments resolved five molecular biomarkers, the underlying architecture is inherently scalable. The multiplexing capacity of this platform could be expanded by integrating additional excitation wavelengths, extending spectral detection, and incorporating complementary contrast mechanisms. These may include Raman-based probes with narrow spectral features (47, 48) as well as label-free modalities such as multiphoton excitation and harmonic generation (15, 49, 50) and nonlinear Raman imaging (51, 52), enabling higher-dimensional molecular phenotyping *in vivo*. Advances in probe brightness, detector sensitivity, and spectral modeling may further increase the number of distinguishable molecular signatures within physiologically achievable photon budgets. Together, these opportunities suggest that hyperspectral microendoscopy could evolve toward higher-dimensional molecular imaging of tumor microenvironments while preserving the video-rate acquisition required for *in vivo* applications.

Collectively, these results demonstrate that photon-calibrated multiplexed spectral microendoscopy enables quantitative, longitudinal mapping of tumor and immune phenotypes at cellular resolution *in vivo*. By bridging microscale molecular imaging with repeated access to intact lesions, SMIRC provides a platform for studying tumor heterogeneity and immune dynamics in preclinical models and may inform future image-guided strategies for therapeutic modulation of the tumor microenvironment.

## Supplementary Material

Supplementary Files

This is a list of supplementary files associated with this preprint. Click to download.

- SupplementaryVideo1.mp4
- 05272026NatCommunicsupplementarymaterialsrev0.pdf

## Data Availability

All data needed to evaluate the conclusions in this paper are present in the paper and/or the Supplementary Materials. Representative hyperspectral imaging datasets underlying the main figures, including raw spectral image cubes, spectrally unmixed images, and mosaic reconstructions used for spatial heterogeneity and power spectral analyses, will be deposited in a public repository and made publicly available upon publication. Owing to the large size (gigabyte-scale) of the complete hyperspectral datasets, additional raw data are available from the corresponding author upon reasonable request. Analysis code for spectral unmixing, mosaicking, and power spectral analysis will be made publicly available upon publication. Software and scripts developed for this study are available at https://github.com/springlabnu/hyperviewer2 (spectral decomposition) and https://github.com/springlabnu/MultiMosaic and https://github.com/springlabnu/Multi-Channel-Mosaicking (micro-image mosaicking), and are distributed under the GNU General Public License version 3 (GPLv3).
